# Associations of Clinical Factors and Blood Groups With the Severity of COVID-19 Infection in Makkah City, Saudi Arabia

**DOI:** 10.3389/fcimb.2022.870096

**Published:** 2022-06-21

**Authors:** Nashwa Shesha, Sami Melebari, Saad Alghamdi, Bassem Refaat, Hind Naffadi, Khalid Alquthami

**Affiliations:** ^1^Serology and Immunology Department, Hera General Hospital, Makkah Healthcare Cluster, Makkah, Saudi Arabia; ^2^Molecular Biology Department, The Regional Laboratory, Ministry of Health, Makkah, Saudi Arabia; ^3^Laboratory Medicine Department, Faculty of Applied Medical Sciences, Umm Al-Qura University, Makkah, Saudi Arabia; ^4^Medical Genetics Department, Faculty of Medicine, Umm Al-Qura University, Makkah, Saudi Arabia; ^5^Molecular Biology Department, Al-Noor Specialist Hospital, Makkah Healthcare Cluster, Makkah, Saudi Arabia

**Keywords:** ABO blood groups, Rh blood groups, COVID-19, Makkah, Western region, Saudi Arabia, risk factors

## Abstract

**Context:**

The possible associations between the different blood groups and clinical factors with COVID-19 infection among patients in Makkah city.

**Objective:**

To investigate the relationship between ABO blood groups and COVID-19 infection in patients who were tested positive and to elucidate the most common ABO blood groups with a higher infectivity of COVID-19 and disease association.

**Materials and Methods:**

This was an observational cross-sectional study that included COVID-19 patients diagnosed with PCR and who were hospitalized in Al-Noor Specialist Hospital (Makkah) during the period between March to November 2020. The ABO and Rhesus blood groups alongside the clinical characteristics were determined and retrieved from medical records and HESN of the Ministry of Health of the Kingdom of Saudi Arabia (KSA).

**Results:**

The overall confirmed COVID-19 cases included in this study were 1,583 patients who underwent positive PCR testing between March and November 2020. The frequencies of blood groups were as follows: group O^+^ (37%), group A^+^ (29.2%), group B^+^ (22.6%), group AB^+^ (5.1%), group O^-^ (2.8%), group B^-^ (1.8%), group A^-^ (1.1%), and group AB^-^ (0.4%). However, no significant correlations were observed for ABO groups and Rh types with the severity of COVID-19 illness. Conversely, signs and symptoms of respiratory distress syndrome (RDS), pneumonia, and respiratory failure symptoms, alongside a history of diabetes mellitus, hypertension, chronic kidney diseases, and congestive heart failure significantly increased the risk of death from COVID-19 infection. Moreover, the rates of fever, cough, and asthma were markedly lower in the deceased group compared with the recovered group of patients.

**Conclusion:**

The association between the different blood groups with the prevalence and mortality of COVID-19 among infected patients has yet to be elucidated as we found no significant differences in the observed versus expected distribution of ABO phenotypes among the included cases. The prevalence of RDS, pneumonia, and respiratory failure was found higher among hospitalized COVID-19 patients in the deceased group. However, other factors such as fever, cough, and asthma appeared to be more significantly lower than in the recovered group.

## Background

Since the first discovery of Coronavirus disease (COVID-19) in December 2019 in Wuhan, China, the virus has been found to cause a severe acute respiratory infection. Coronavirus is a strain of beta coronavirus that belongs to the coronaviridae family which is classified based on phylogenetic relationship and genomic structure into SARS-COV, SARS-COV2 (COVID-19), and MERS-COV (Payne, 2017).

The total number of reported cases of COVID-19 is over 270 million confirmed infections with total deaths exceeding five million across the globe as of December 13, 2021 (Control, E.C.f.D.P.a., 2020). In March 2020, the number of positive cases in the kingdom of Saudi Arabia (KSA) increased exponentially and advocated a potential outbreak within the community. The corresponding figures for KSA were 347,282 with the total death of 5,402 respectively as of November 1, 2020 (Control, E.C.f.D.P.a., 2020). However, the total confirmed cases by December 17, 2021, exceeded 551,000 across the kingdom with 8,856 deaths (Control, E.C.f.D.P.a., 2020).

Concerns related to infection persistence, as well as the effectiveness of the available vaccines against viral spread, have been raised following the detection of mutations in SARS-COV2 genome that introduced several highly infective viral genotypes. However, many risk factors contribute to the infectiveness of COVID-19, including patients’ age, gender, and medical history. In addition, some evidence suggests that genetic factors like ABO and Rh blood groups would enhance patient susceptibility and influence the occurrence of COVID-19 viral infection. Different type of blood groups can interact with several pathogens which can play a role in the immune response and cell signalling (Cooling, 2015)

ABO blood group antigens (A, B and H) are present on erythrocytes and they are made on the surface of erythropoietic precursor cells. ABO blood group is classified into four basic ABO phenotypes which are identified as A, B, AB, and O (Franchini and Bonfanti, 2015). A previous report has suggested a possible correlation between ABO blood type and susceptibility to COVID-19, as it showed that ABO blood groups are correlated with the risk of developing the disease, its severity, and disease behavior among patients infected with COVID-19 (Cheng et al., 2005).

A study of Zhao et al. has disclosed that in Wuhan, China, the population of A blood type were more likely to be affected by COVID-19 than the people with O blood type who were found less likely to be affected (Zhao et al., 2020). Similarly, data from Italy and Spain suggested higher incidence of COVID-19 in patient with blood group A as compared with group O which could be linked to the presence of natural anti-blood group antibodies (Ellinghaus et al., 2020). Moreover, a recent study in Qatif, KSA revealed a significantly lower risk for O blood group among COVID-19 patients while the patients with AB blood group have higher susceptibility (Aljanobi et al., 2020).

Interestingly, another study in the Western province in Jeddah, KSA, showed that COVID-19 infection was more frequent in blood group O (50.1%), while blood group AB disclosed the lowest rate (4.1%), among 35,388 Saudi blood donors (Alzahrani et al., 2018). A recent review by Kabrah et al. also reported that individuals with blood group A had significantly higher risks, whereas blood type AB showed lower risks, of acquiring COVID-19 infection (Kabrah et al., 2021). However, diverse populations and different geographical locations could be attributed to the susceptibility of COVID-19 infection.

The transmission dynamics and mechanisms of COVID-19 have been suggested to elucidate the link between the different blood groups and their effect on infection susceptibility. The presence of anti-A antibodies in blood group O and B has been suggested to play a role in inhibiting viral adhesion to the host cells by blocking the ACE 2 receptor, which is the main gate for COVID-19 entry into the cell (Gérard et al., 2020). Moreover, the higher rates of COVID-19 infection in individuals with blood group O could be related to lower plasma levels of the von Willebrand factor (vWF) and factor VIII (FVIII) compared with the other blood groups (Yamamoto et al., 2021).

All these findings could acknowledge the significance of blood group phenotypes in the acquisition and progression of COVID-19 infection. Hence, identification of factors involved in the occurrence of COVID-19 infection could aid in the development of new strategies for estimating the spread and severity of infection. However, there is a shortage of data showing the relationship of different blood groups with the pathogenesis of COVID-19 in the Makkah region of the KSA. Therefore, and to the best of our knowledge, this study is the first to measure the associations of several factors in addition to ABO blood groups with the rates and severity of COVID-19 infection among a local population in Makkah city, KSA.

## Materials and Methods

### Ethical Approval

This study was approved by the local ethical committee of the Directorate of Health Affairs in Makkah (H-02-K-076-1120-406).

### Study Design and Patients

This was a retrospective study that included COVID-19 patients admitted between March and November 2020 to Al-Noor Specialist Hospital in Makkah city, KSA, and who were diagnosed by PCR using nasopharyngeal swabs. During the study period, the total numbers of positive COVID-19 cases in KSA were 347,282 among whom 5,402 died from infection (Control, E.C.f.D.P.a., 2020). The Epi Info™ software was used (https://www.cdc.gov/epiinfo/index.html), and the minimum required sample size was 583 participants to achieve a study power of 95%.

### Study Samples

Suspected COVID-19 cases were examined, and infection was confirmed using the novel coronavirus (2019-nCoV) detection qPCR kit at Makkah Regional laboratory and by following the manufacturer’s instructions. Briefly, all samples were initially screened for the Sarbecovirus subgenus envelope (E) gene and the positive samples were then confirmed by detecting the RdRP or ORFa1b viral genes. Patients with negative results were excluded from the study. The severity of infection was categorized as mild, moderate, and severe if the patients were admitted to emergency room (ER), medical ward, and ICU, respectively. The written informed consent was waived since information identifying the patients were not included in the collected data.

Demographic data related to age, gender, and nationality alongside ABO and Rh blood groups, disease prognosis (recovered/dead), and medical history, such as diabetes mellitus, kidney disease, hypertension, and congestive heart failure (HF) were collected. Additionally, the clinical features associated with COVID-19 infection, including fever, cough, dyspnoea, pneumonia, asthma, respiratory distress syndrome (RDS), and respiratory failure were included. All data were retrieved from the health electronic surveillance system (HESN).

### Statistical Analysis

The patients were categorized according to prognosis either as recovered or deceased and the distribution of the different categorial variables were compared between both groups by cross-tabulation followed by Chi-square (χ^2^) test using SPSS version 25. Crude odds ratio with 95% confidence interval (CI) were also measured for each variable. Binary logistic regression analysis was also done to identify the factors associated with the severity of COVID-19. P < 0.05 was considered statistically significant.

## Results

### Overall Demographic and Clinical Characteristics of COVID-19 Patients

A total of 1,583 patients with confirmed COVID-19 infection by PCR alongside a complete final status (recovered or deceased) between March and November 2020 were included in the study. The overall mean age of the 1,583 patients was 50.8 ± 15.8 years and the numbers of patients with ≤ 50 years of age 784 (49.5%) and those with age > 50 years 799 (50.5%) were equal. The numbers of diagnosed cases were 272 (17.2%) between March and May, 1,148 (72.5%) between June and August, and 163 (10.3%) between September and November 2020. Moreover, male (n = 978; 61.8%) and non-Saudi (n = 865; 54.6%) patients were more prevalent in the study cohort. Most of the patients were admitted to medical wards (n = 1214; 46.7%), while the remainder was admitted to the ER (n = 212; 13.4%) or ICU (n = 157; 9.9%), and the overall median duration of hospitalization was four days (IQR: 2 – 9 days).

Most common recorded symptoms at the time of presentation were pneumonia 408 (25.8%), fever 323 (20.4%), dyspnoea 294 (18.6%), cough 293 (18.5%), respiratory distress syndrome 79 (5%), and respiratory failure 66 (4.2%). Based on the medical history, 303 patients had diabetes (19.1%), 193 had hypertension (12.2%), 185 had chronic kidney disease (11.7%), 69 had congestive heart failure (4.4%), and 36 cases (2.3%) had a history of asthma. Additionally, the frequencies of blood groups among the 1,583 patients in descending order were as follows: O^+^ 686 (37%), A^+^ 463 (29.2%), B^+^ 357 (22.6%), AB^+^ 80 (5.1%), O^-^ 44 (2.8%), B^-^ 28 (1.8%), A^-^ 18 (1.1%) and AB^-^ 7 (0.4%).

### Demographic and Clinical Characteristics of COVID-19 Patients According to Demise

A total of 178 patients (11.2%) with COVID-19 infection died during the study period and the mean age was significantly higher in the deceased group (**Table 1**). Moreover, death occurred in 34.7% (n = 70/272) of patients diagnosed between March and May, 6.4% (n = 74/1148) of cases diagnosed between June and August, and 20.9% (n = 34/163) of patients diagnosed between September and November 2020. By further analysis, significantly longer hospitalization durations and higher rates of ICU admission were detected in the deceased group (**Table 1**). Concomitantly, the deceased group had markedly higher frequencies of RDS, pneumonia and respiratory failure symptoms alongside history of diabetes mellitus, hypertension, chronic kidney disease, and congestive heart failure, while the rates of fever, cough, and asthma were markedly lower than the recovered group (**Table 1**). Additionally, being older than 50 years, staying more than 7 days in the hospital, showing symptoms of RDS, pneumonia, and respiratory failure, together with having history of diabetes mellitus, hypertension, chronic kidney disease, and congestive heart failure were associated with significantly higher crud odds ratio of death from COVID-19 infection (**Table 1**). Conversely, there was no significant difference between the recovered and deceased groups in the distribution of each of the blood groups (**Table 1**).

**Table 1:** The demographic and clinical characteristics of the study participants (n = 1583) with crud odds ratio according to demise.

Parameter		COVID-19 positive cases (n = 1583; 100%)		P value	Crud Odds Ratio (95% CI)
		Recovered (n = 1405; 88.8%)	Deceased (n = 178; 11.2%)		
**Mean ± SD of Age (year)**		49.5 ± 15.4	60.5 ± 15.5	**P < 0.0001**	N/A
**Age groups**	≤ 50 Years > 50 Years	741 (46.8%) 664 (42%)	43 (2.7%) 135 (8.5%)	**P < 0.0001**	**Ref.** **3.504 (2.447 – 5.016)**
**Month of admission**	March-May June-August September-November	202 (12.8%) 1074 (67.8%) 129 (8.2%)	70 (4.4%) 74 (4.7%) 34 (2.1%)	**P < 0.0001**	N/A
**Type of Admission**	Ward ER ICU	1161 (73.3%) 180 (11.4%) 64 (4.1%)	53 (3.3%) 32 (2%) 93 (5.9%)	**P < 0.0001**	N/A
**Median days of hospitalization (IQR)**		4 (2 – 7)	13 (7 – 18)	**P < 0.0001**	N/A
**Hospitalisation Duration groups**	≤ 7 Days > 7 Days	1055 (66.8%) 346 (22%)	48 (3%) 130 (8.2%)	**P < 0.0001**	**Ref.** **8.258 (5.804 – 11.749)**
**Gender**	Male Female	876 (55.4%) 529 (33.4%)	102 (6.4%) 76 (4.8%)	0.1	Ref. 1.234 (0.900 – 1.692)
**Nationality**	Saudi Non-Saudi	626 (39.6%) 779 (49.2%)	92 (5.8%) 86 (5.4%)	0.07	Ref. 1.331 (0.974 – 1.819)
**Fever**	No Yes	1088 (75.1%) 317 (20.1%)	172 (10.9%) 6 (0.3%)	**P < 0.0001**	**Ref.** **0.120 (0.053 – 0.273)**
**Cough**	No Yes	1188 (68.7%) 287 (18.1%)	172 (10.9%) 6 (0.3%)	**P < 0.0001**	**Ref.** **0.136 (0.061 – 0.310)**
**Dyspnoea**	No Yes	1112 (70.3%) 293 (18.5%)	177 (11.1%) 1 (0.1%)	**P < 0.0001**	**Ref.** **0.021 (0.003 – 0.154)**
**Pneumonia**	No Yes	1071 (67.7%) 334 (21.1%)	104 (6.5%) 74 (4.7%)	**P < 0.0001**	**Ref.** **2.282 (1.653 – 3.150)**
**Asthma**	No Yes	1357 (85.7%) 30 (1.9%)	172 (10.9%) 6 (0.3%)	0.3	Ref. 1.599 (0.656 – 3.896)
**Respiratory distress Syndrome**	No Yes	1401 (88.5%) 4 (0.3%)	103 (6.5%) 75 (4.7%)	**P < 0.0001**	**Ref.** **255.03 (91.462 – 711.156)**
**Respiratory Failure**	No Yes	1376 (86.9%) 29 (1.9%)	141 (8.9%) 37 (2.3%)	**P < 0.0001**	**Ref.** **12.451 (7.432 – 20.861)**
**Diabetes Mellitus**	No Yes	1174 (74.2%) 231 (14.6%)	106 (6.7%) 72 (4.5%)	**P < 0.0001**	**Ref.** **3.452 (2.480 – 4.806)**
**Kidney Diseases**	No Yes	1317 (83.2%) 88 (5.6%)	106 (5.1%) 97 (6.1%)	**P < 0.0001**	**Ref.** **17.922 (12.435 – 25.830)**
**Hypertension**	No Yes	1269 (80.2%) 136 (8.6%)	121 (7.6%) 57 (3.6%)	**P < 0.0001**	**Ref.** **4.396 (3.063 – 6.307)**
**Congestive heart failure**	No Yes	1370 (86.6%) 34 (2.2%)	143 (9%) 35 (2.2%)	**P < 0.0001**	**Ref.** **9.862 (5.967 – 16.299)**
**Blood group**	A- A+ B- B+ AB- AB+ O- O+	17 (1.1%) 403 (25.4%) 26 (1.6%) 324 (20.5%) 6 (0.4%) 73 (4.6%) 39 (2.5%) 517 (32.7%)	1 (0.1%) 60 (3.8%) 2 (0.1%) 33 (2.1%) 1 (0.1%) 7 (0.4%) 5 (0.3%) 69 (4.3%)	0.7	N/A

The significant values are in bold.

Furthermore, the binary logistic regression data revealed that the risk of death from COVID-19 infection increased significantly with older age (3%), ICU admission (> 6-fold), > 7 days of hospitalization (> 2-fold), symptoms of RDS (76-fold) or respiratory failure (7-fold), history of chronic kidney diseases (> 7-fold), congestive heart failure (3.8-fold) and hypertension (1.7-fold; **Table 2**).

**Table 2:** The associations of demographic and clinical characteristics of COVID-19 patients with mortality from infection by binary logistic regression analysis.

Predictors	Death from COVID-19 Infection	
	Odds ratio (95%CI)	P Value
Age (year)	1.030 (1.002 – 1.059)	P = 0.03
Age groups ≤ 50 Years > 50 Years	Ref. 1.064 (0.414 – 2.734)	NS
Month of Admission March-May 2020 June-August 2020 September-November 2020	Ref. 0.425 (0.220 – 0.824) 1.340 (0.579 – 3.101)	P = 0.01 NS
Type of Admission ER Ward ICU	Ref. 0.353 (0.158 – 0.790) 6.805 (2.860 – 16.193)	P = 0.01 P < 0.0001
Hospitalisation duration (days)	1.024 (0.985 – 1.064)	NS
Hospitalisation duration groups ≤ 7 Days > 7 Days	Ref. 2.361 (1.072 – 5.199)	P = 0.03
Fever No Yes	Ref. 0.268 (0.056 – 1.283)	NS
Cough No Yes	Ref. 1.625 (0.432 – 6.108)	NS
Dyspnoea No Yes	Ref. 0.034 (0.003 – 0.399)	P = 0.007
Pneumonia No Yes	Ref. 1.340 (0.737 – 2.438)	NS
Respiratory distress Syndrome No Yes	Ref. 76.847 (23.744 – 248.716)	P < 0.0001
Respiratory Failure No Yes	Ref. 7.055 (3.018 – 16.492)	P < 0.0001
Diabetes Mellitus No Yes	Ref. 1.695 (0.868 – 3.310)	NS
Kidney Diseases No Yes	Ref. 7.251 (3.852 – 13.649)	P < 0.0001
Hypertension No Yes	Ref. 1.729 (0.816 – 3.662)	NS
Congestive heart failure No Yes	Ref. 3.781 (1.532 – 9.331)	P = 0.004

^*NS, non-significant.^

## Discussion

COVID-19 is a serious infectious disease caused by the novel severe acute respiratory syndrome coronavirus 2, which became a pandemic a few weeks after the first case was reported in Wuhan, China during late December 2019 (Alinia-Ahandani and Sheydaei, 2020). In patients infected with SARS-CoV-2, the disease severity ranges from asymptomatic infection to severe illness and in some cases, death. Since this is a novel virus, limited information is available about the risk factors associated with infection severity, as well as death, in the different populations. Nonetheless, the most commonly identified factors increasing the risk of death in COVID-19 patients include hypertension, diabetes, male gender, older age, and individuals with immunosuppression status (Wu et al., 2020; Zhou et al., 2020). However, the death rate per million population from COVID-19 infection varied greatly between countries. For instance, the United Kingdom had much higher COVID-19 deaths per million population as compared to Malaysia (Allaham et al., 2021). Several other factors may also influence COVID-19 deaths, including age, genetic factors, COVID-19 diagnosis rates, as well as health care provision scheme in each country. Therefore, it is important to conduct more epidemiological studies in the different societies to fully understand the disease natural history and its associated relevant risk factors.

In this paper we carried out a retrospective analysis to identify the risk factors for death in hospitalized COVID-19 patients during the early wave of the pandemic and before the introduction of vaccines (March to November 2020). Out of the 1,583 patients admitted to Al Noor Specialist Hospital in Makkah city, 1,405 patients recovered and 178 died. The comparison of patient’s characteristics between the recovered and deceased patients revealed several factors that contributed to death from infection.

Firstly, we divided into three-time intervals according to the time of diagnosis and admission as follows: March-May, June-August, and September-November seasonal quarters. The rates of admission were significantly different between the three quarters and the highest rates of admission were seen during June-August showing, which corresponds to the time of stringent lock-down regulations applied by the authorities in the KSA (Shimul et al., 2021). Nonetheless, the observed rates of infection are much lower than those reported by other countries during the same period, including the United States of America (Dukhovnov and Barbieri, 2021) and United Kingdom (Flynn et al., 2020). Moreover, the frequencies of death between June and August were equal to those recorded in the first quarter, suggesting that the applied curfew regulations could have limited the severity of COVID-19 infection (Shimul et al., 2021; Alswaidi et al., 2021).

The rate of recovery from infection was significantly higher in patients younger than 50 years, which correlates with the findings of an earlier study from Wuhan in China (Wu et al., 2020). A possible explanation could be related to age-dependent complications along with weakened immune systems in elders that could contribute to death from COVID-19 infection (Wu et al., 2020). Moreover, our data shows that the rates of infection were significantly higher, whereas the frequencies of death were lower in non-Saudis (49.2% recovered and 5.4% deceased) as compared to Saudis (39.6% recovered and 5.8% deceased). Expatriates account for 26% of the population in Saudi Arabia, and many of them live in shared dorms, which could contribute to a wider spread of infection (Alswaidi et al., 2021). Conversely, the non-Saudi patients were significantly lower in age compared with Saudis, which could provide an explication for the observed higher rates of recovery from COVID-19 infection in the former group (Wu et al., 2020).

Fever, cough, symptoms of respiratory distress syndrome, and respiratory failure were the most prevalent symptoms reported by the study participants and significantly increased the risk of death from COVID-19 infection. The present observations agree with the findings of several earlier studies that have shown increased risk of death in COVID-19 patients presented with acute symptoms and/or signs of respiratory distress (Zhou et al., 2020; Alsofayan et al., 2020; Chen et al., 2020). Additionally, history of diabetes mellitus, kidney diseases, congestive heart failure, and hypertension significantly increased the risk of death from COVID-19 in the current report, which correlates with many prior population-based studies that have reported these co-morbidities as major risk factors for death from COVID-19 (Alsofayan et al., 2020; Alguwaihes et al., 2020; Varikasuvu et al., 2021). Indeed the co-existence of chronic diseases with COVID-19 infection could compromise immunity and hemodynamic stability, thus worsening the prognosis of COVID-19 infection (Andrade et al., 2021; Dessie and Zewotir, 2021). Additionally, patients with heart failure are at a particularly high risk of COVID-19 death and earlier coronavirus and influenza outbreaks were shown to worsen a pre-existing heart failure by numerous mechanisms (Bader et al., 2021).

The rates of COVID-19 infection were substantially higher in blood groups O+ (37%), A^+^ (29.2%) and B^+^ (22.6%), while the AB^-^ group was the least frequent group in the study participants (0.4%). The present report correlates with a prior study from the same province in KSA that had shown higher rates of infection in individuals with blood group O (Alzahrani et al., 2018)., which could be due to lower serum concentrations of von Willebrand factor (vWF) and factor VIII (FVIII) in this group (Yamamoto et al., 2021). However, there was no associations between the different blood groups and the risk of death from COVID-19 infection in the present study. Collectively, our data and the earlier reports suggest that blood group O could increase the risk of infection, but not death, from COVID-19. In contrast, others demonstrated higher rates of COVID-19 infection in patients with blood group A, whereas blood group O appeared to be protective (Zhao et al., 2020; Ellinghaus et al., 2020; Aljanobi et al., 2020; Kabrah et al., 2021). Hence, additional studies are still needed to precisely measure the roles of blood groups in COVID-19 infection.

While interpreting the results of this study, it may be necessary to consider several limitations. Since this study was retrospective and cross-sectional, clinical courses and outcomes could not be fully assessed as we analyzed the past data. Further studies of COVID-19 in the region are advised to elaborate on severity predications since the study location (Al Noor Specialist Hospital) is a secondary care institution and these findings may not be reflective of the milder COVID-19 infections. Patients with milder disease tend to go to smaller clinics and hospitals.

## Conclusion

This study measured several factors that could have increased the risk of death from COVID-19 infection in the Western Province of Saudi Arabia during the early waves of the pandemic. COVID-19 related symptoms such as fever, cough, pneumonia, and respiratory distress syndrome were the most frequently reported and significantly increased the risk of death in our cohort of patients. In addition, history of renal and cardiovascular diseases contributed to the severity and prognosis of COVID-19 infection and were associated with higher mortality rates, highlighting the importance of early diagnosis and supportive care in such patients. Although the frequencies of blood groups O+, A^+^ and B^+^ were markedly higher in our study population, the distributions of the different blood groups were not associated with increased risk of death from COVID-19. Therefore, more studies with larger populations are mandatory to measure the correlations between blood groups and the severity of COVID-19 infection.

## Data Availability Statement

The original contributions presented in the study are included in the article/supplementary material. Further inquiries can be directed to the corresponding author.

## Ethics Statement

The studies involving human participants were reviewed and approved by IRB local ethical committee number (H-02-K-076- 1120-406). Written informed consent for participation was not required for this study in accordance with the national legislation and the institutional requirements.

## Author Contributions

NS and SM contributed to the conception and design of the study. SA collected the data and NS organized it. BR performed the statistical analysis. NS, SM, BR, and SA wrote the first draft of the manuscript and reviewed it. SA and HN reviewed all manuscript sections. All authors contributed to manuscript revision, read, and approved the submitted version.

## Conflict of Interest

The authors declare that the research was conducted in the absence of any commercial or financial relationships that could be construed as a potential conflict of interest.

## Publisher’s Note

All claims expressed in this article are solely those of the authors and do not necessarily represent those of their affiliated organizations, or those of the publisher, the editors and the reviewers. Any product that may be evaluated in this article, or claim that may be made by its manufacturer, is not guaranteed or endorsed by the publisher.

## References

[B23] Alguwaihes A. M. Al-Sofiani M. E. Megdad M. Albader S. S. Alsari M. H. Alelayan A.. (2020). Diabetes and Covid-19 Among Hospitalized Patients in Saudi Arabia: A Single-Centre Retrospective Study. Cardiovasc. Diabetol. 19 (1), 205. doi: 10.1186/s12933-020-01184-4 33278893 PMC7718833

[B13] Alinia-Ahandani E. Sheydaei M. (2020). Overview of the Introduction to the New Coronavirus (Covid19): A Review. J. Med. Biol. Sci. Res. 6(2): 14–20. doi: 10.36630/jmbsr_20005

[B8] Aljanobi G. A. Alhajjaj A. H. Alkhabbaz F. L. Al-Jishi J. M. (2020). The Relationship Between ABO Blood Group Type and the COVID-19 Susceptibility in Qatif Central Hospital, Eastern Province, Saudi Arabia: A Retrospective Cohort Study. Open J. Internal Med. 10 (2), 232–238.doi: 10.4236/ojim.2020.102024

[B16] Allaham S. Demel I. C. Nur I. Abu Salim F. N. Manikam L. (2021). The Impact of United Kingdom and Malaysia’s Inherent Health Systems on Their COVID-19 Responses: A Comparison of Containment Strategies. World Med. Health Policy 4, 10.1002/wmh3.412. doi: 10.1002/wmh3.412 PMC8242468 34226852

[B21] Alsofayan Y. M. Althunayyan S. M. Khan A. A. Hakawi A. M. Assiri A. M. (2020). Clinical Characteristics of COVID-19 in Saudi Arabia: A National Retrospective Study. J. Infect. Public Health 13 (7), 920–925. doi: 10.1016/j.jiph.2020.05.026 32534945 PMC7833063

[B20] Alswaidi F. M. Assiri A. M. Alhaqbani H. H. Alalawi M. M. (2021). Characteristics and Outcome of COVID-19 Cases in Saudi Arabia: Review of Six-Months of Data (March-August 2020). Saudi Pharm. J. 29 (7), 682–691. doi: 10.1016/j.jsps.2021.04.030 34400862 PMC8347652

[B9] Alzahrani F. M. Shaikh S. S. Rasheed M. A. (2018). Frequency of ABO-Rhesus Blood Groups in the Western Region of Saudi Arabia. J. King Abdulaziz Univ. - Med. Sci. 25 (1), 9–13. doi: 10.4197/med.25-1.2

[B25] Andrade J. A. Muzykovsky K. Truong J. (2021). Risk Factors for Mortality in COVID-19 Patients in a Community Teaching Hospital. J. Med. Virol. 93 (5), 3184–3193. doi: 10.1002/jmv.26885 33595120 PMC8014291

[B2] Control, E.C.f.D.P.a. (2020). Available at: https://www.ecdc.europa.eu/en/covid-19-pandemic.

[B27] Bader F. Manla Y. Atallah B. Starling R. C. (2021). Heart Failure and COVID-19. Heart Fail Rev. 26 (1), 1–10. doi: 10.1007/s10741-020-10008-2 32720082 PMC7383122

[B22] Chen T. Wu D. Chen H. Yan W. Yang D. Chen G.. (2020). Clinical Characteristics of 113 Deceased Patients With Coronavirus Disease 2019: Retrospective Study. BMJ 368, m1091. doi: 10.1136/bmj.m1091 32217556 PMC7190011

[B5] Cheng Y. Cheng G. Chui C. H. Lau F. Y. Chan P. K. Ng M. H.. (2005). ABO Blood Group and Susceptibility to Severe Acute Respiratory Syndrome. JAMA 293 (12), 1450–1451. doi: 10.1001/jama.293.12.1450-c 15784866

[B3] Cooling L. (2015). Blood Groups in Infection and Host Susceptibility. Clin. Microbiol. Rev. 28 (3), 801–870. doi: 10.1128/CMR.00109-14 26085552 PMC4475644

[B26] Dessie Z. G. Zewotir T. (2021). Mortality-Related Risk Factors of COVID-19: A Systematic Review and Meta-Analysis of 42 Studies and 423,117 Patients. BMC Infect. Dis. 21 (1), 855. doi: 10.1186/s12879-021-06536-3 34418980 PMC8380115

[B18] Dukhovnov D. Barbieri M. (2021). County-Level Socio-Economic Disparities in COVID-19 Mortality in the USA. Int. J. Epidemiol. 27, dyab267. doi: 10.1093/ije/dyab267 PMC8755340 34957523

[B7] Ellinghaus D. Degenhardt F. Bujanda L. Buti L. Albillos A. Invernizzi P.. (2020). Genomewide Association Study of Severe Covid-19 With Respiratory Failure. N. Engl. J. Med. 383 (16), 1522–1534. doi: 10.1056/NEJMoa2020283 32558485 PMC7315890

[B19] Flynn D. Moloney E. Bhattarai N. Scott J. Breckons M. Avery D.. (2020). COVID-19 Pandemic in the United Kingdom. Health Policy Technol. 9 (4), 673–691. doi: 10.1016/j.hlpt.2020.08.003 32874853 PMC7451057

[B4] Franchini M. Bonfanti C. (2015). Evolutionary Aspects of ABO Blood Group in Humans. Clin. Chim. Acta 444, 66–71. doi: 10.1016/j.cca.2015.02.016 25689219

[B11] Gérard C. Maggipinto G. Minon J.-M. (2020). COVID-19 and ABO Blood Group: Another Viewpoint. Br. J. Haematol. 190 (2), e93–e94. doi: 10.1111/bjh.16884 32453863 PMC7283642

[B10] Kabrah S. M. Kabrah A. M. Flemban A. F. Abuzerr S. (2021). Systematic Review and Meta-Analysis of the Susceptibility of ABO Blood Group to COVID-19 Infection. Transfus. Apheresis Sci. 60 (4), 103169. doi: 10.1016/j.transci.2021.103169 PMC8139534 34045120

[B1] Payne S. (2017). Family Coronaviridae. Viruses p, 149–158. doi: 10.1016/B978-0-12-803109-4.00017-9

[B17] Shimul S. Alradie-Mohamed A. Kabir R. Al-Mohaimeed A. Mahmud I. (2021). Effect of Easing Lockdown and Restriction Measures on COVID-19 Epidemic Projection: A Case Study of Saudi Arabia. PLoS One 16 (9), e0256958. doi: 10.1371/journal.pone.0256958 34499681 PMC8428777

[B24] Varikasuvu S. R. Dutt N. Thangappazham B. Varshney S. (2021). Diabetes and COVID-19: A Pooled Analysis Related to Disease Severity and Mortality. Prim. Care Diabetes 15 (1), 24–27. doi: 10.1016/j.pcd.2020.08.015 32891525 PMC7456278

[B14] Wu C. Chen X. Cai Y. Xia J. Zhou X. Xu S.. (2020). Risk Factors Associated With Acute Respiratory Distress Syndrome and Death in Patients With Coronavirus Disease 2019 Pneumonia in Wuhan, China. JAMA Intern. Med. 180 (7), 934–943. doi: 10.1001/jamainternmed.2020.0994 32167524 PMC7070509

[B12] Yamamoto F. Yamamoto M. Muñiz-Diaz E. (2021). Blood Group ABO Polymorphism Inhibits SARS-CoV-2 Infection and Affects COVID-19 Progression. Vox Sang. 116 (1), 15–17. doi: 10.1111/vox.13004 32965025 PMC7537232

[B6] Zhao J. Yang Y. Huang H. Li D. Gu D. Lu X.. (2020). Relationship Between the ABO Blood Group and the COVID-19 Susceptibility. medRxiv 73 (2), 328–331. doi: 10.1093/cid/ciaa1150 PMC7454371 32750119

[B15] Zhou F. Yu T. Du R. Fan G. Liu Y. Liu X.. (2020). Clinical Course and Risk Factors for Mortality of Adult Inpatients With COVID-19 in Wuhan, China: A Retrospective Cohort Study. Lancet 395 (10229), 1054–1062. doi: 10.1016/S0140-6736(20)30566-3 32171076 PMC7270627

